# Complication rates and reduction potential of palmar versus dorsal locking plate osteosynthesis for the treatment of distal radius fractures

**DOI:** 10.1007/s10195-014-0306-y

**Published:** 2014-07-16

**Authors:** F. Wichlas, N. P. Haas, A. Disch, D. Machó, S. Tsitsilonis

**Affiliations:** 1 Center for Musculoskeletal Surgery, Charité,Universitätsmedizin Berlin, Augustenburger Platz 1, 13353 Berlin, Germany; 2 Berlin-Brandenburg Center of Regenerative Therapies, Charité,Universitätsmedizin Berlin, Berlin, Germany

**Keywords:** Distal radius fracture, Locking plate, Approach, Complication

## Abstract

**Background:**

The aim of this study was to evaluate the complication rates of volar versus dorsal locking plates and postoperative reduction potential after distal radius fractures.

**Materials and methods:**

For this study 285 distal radius fractures (280 patients/59.4 % female) treated with locked plating were retrospectively evaluated. The mean age of the patients was 54.6 years (SD 17.4) and the mean follow-up was 33.2 months (SD 17.2). The palmar approach was used in 225 cases and the dorsal approach in 60 cases (95 % type C fractures).

**Results:**

Adequate reduction was achieved with both approaches, regardless of fracture severity. In the dorsal group, the complications and implant removal rates were significantly higher and the operative time was also longer.

**Conclusions:**

Based on these facts, we advocate the palmar locking plate for the vast majority of fractures. In cases of complex multifragmentary articular fractures where no compromise in reduction is acceptable, and with the biomechanical equality of palmar and dorsal plating remaining unproven, dorsal plating may still be considered.

**Level of evidence:**

Therapeutic level IV.

## Introduction

Over recent years an increase in the operative treatment of distal radius fractures has been observed [1]. Despite this increase and the high incidence of distal radius fractures, several facts have not yet been fully elucidated, especially in terms of surgical approach and complication rates. The biomechanical advantages of locking plates over the traditional plates have resulted in an increase of volar plating [2]. Volar plating is considered to be a more straightforward surgical procedure, which can result in anatomic reduction through indirect reduction techniques and plate manipulation; however, dorsal articular fragments cannot be directly visualized and controlled. On the other hand, the dorsal approach is surgically more demanding and is thought to be associated with higher complication rates. Tendon ruptures or tenosynovitis due to exposure of the tendons or implant-associated soft-tissue irritation appears to be more common after the dorsal approach [3]. On these grounds, the introduction of the volar locking plate with the principle of subchondral buttressing of the joint surface substantially questioned the need for dorsal plating. However, an achievement of anatomic reduction after volar plating is not thought to be always possible, especially in the case of complex intra-articular ‘pilon radial’ fractures with central depression fragments and extended dorsal articular comminution. Under this scope, re-evaluation of the indications for volar versus dorsal plating is important when taking complication rates, fracture complexity, and individual patient characteristics into consideration.

The aim of the present study was the evaluation of complication rates of volar and dorsal locking plate osteosynthesis, as well as the evaluation of postoperative radiological fracture reduction.

## Materials and methods

For the needs of the present study all patients with distal radius fractures that were operatively treated with a locking plate (2.4 and 3.5 mm Locking Compression Plate (LCP), Synthes^®^, Oberdorf, Switzerland) over a 3-year period (2005–2007) were included and retrospectively evaluated. All patients gave informed consent prior to being included in the study. The study was authorized by the local ethical committee (EA2/075/11) and was performed in accordance with the Ethical standards of the 1964 Declaration of Helsinki as revised in 2000. An electronic ICD-9 search was conducted and 285 distal radius fractures (280 patients) treated with an LCP were identified. The mean age was 54.6 years (SD 17.4), and the majority of patients were female [116 male (40.6 %)/169 female (59.4 %]. The mean follow-up time was 33.2 months (SD 17.2). A 2.4-mm LCP was used in 192 cases (67.4 %) and a 3.5-mm LCP in 93 cases (32.6 %). The mechanism of injury in the majority of the cases was a fall from standing height (172 cases, 60.5 %). The remaining fractures were caused by sports activities (54 cases, 18.9 %), fall from a greater height (29 cases, 10.1 %), motor vehicle accident (24 cases, 8.3 %), and polytrauma (6 cases, 2.1 %). A palmar approach was used in 225 cases and a dorsal approach in 60 cases. The dorsal approach was used for fractures with a central articular depression or which had dorsal joint fragments that were not considered amendable through a palmar approach. All patients were operated under general anesthesia and operative steps were fluoroscopically controlled under an image intensifier. A perioperative single-shot antibiosis was given and a pneumatic tourniquet was used. The palmar approach was located over the flexor carpi radialis tendon and the dorsal approach located over the third extensor tendon sheath. For the dorsal approach, the retinaculum was opened in a z-shaped way right above the third extensor tendon sheath and the extensor pollicis longus tendon (EPL) was released. An epiperiosteal preparation was conducted medially and laterally. The second plate was placed between the first and second extensor sheath radially. Both approaches are described in detail elsewhere [4]. All fractures except for nine were closed. The evaluated data were fracture classification according to AO, mechanism of injury, operative time, type of implant, peri- and postoperative complications and the need for an implant removal. Fractures were further subdivided into volar and dorsal plate osteosynthesis groups. Fracture reduction was assessed using radial inclination, palmar tilt, and ulnar variance in posteroanterior (PA) and lateral radiographs according to the criteria defined by Kreder et al. [5]. These values were measured pre- and postoperatively.

Continuous variables were expressed as mean ± standard deviation (SD), whereas categorical variables were expressed as percentages (%). The Kolmogorov–Smirnov test was used in order to assess distribution normality. For parametric variables, the Student *t* test was used for the comparison of two groups; for non-parametric variables the Mann–Whitney test was implemented. Differences for categorical variables were assessed with the chi-squared test or Fisher’s exact test. Correlations were examined with either Pearson product moment correlation coefficient or Spearman’s rank correlation coefficient. Differences were considered statistically significant if the null hypothesis could be rejected with >95 % confidence (*p* < 0.05).

## Results

**Fig. 1:**
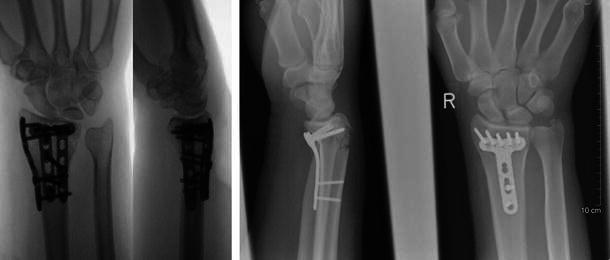
Postoperative x-rays of a dorsal (AO 23 C3) (*left*) and volar locking plate osteosynthesis (AO 23 A3) (*right*)

The fracture distribution according to the AO classification is shown in Table 1. No statistically significant age difference existed between the two groups [mean age of volar group 55.4 years (SD 18.0); mean age of dorsal group 50.7 years (SD 16.3) (*p* = 0.068)]. The dorsal approach group consisted of 95 % (57 fractures) type C fractures, with more than half being (53.3 %) complex C3 fractures. The mean operative time for the volar plating group was 97.3 (SD 42.5) min and 123.7 (SD 49.3) min for the dorsal group. This difference was statistically significant (*p* < 0.001) (Fig. 1).

**Table 1:** Fracture distribution in the study population and in the subgroups according to the AO classification

Type of fracture	Group		
	All (*n* = 285) (%)	Volar (*n* = 225) (%)	Dorsal (*n* = 60) (%)
A	89 (31.2)	82 (36.4)	7 (11.6)
A2	11 (3.8)	11 (4.9)	0 (0)
A3	78 (27.4)	71 (31.5)	7 (11.6)
B	16 (5.6)	16 (7.1)	0 (0)
B2	8 (2.8)	8 (3.5)	0 (0)
B3	8 (2.8)	8 (3.5)	0 (0)
C	180 (63.2)	123 (56)	57 (95)
C1	41 (14.4)	37 (16.4)	4 (6.7)
C2	70 (24.5)	49 (21.8)	21 (35)
C3	69 (24.3)	37 (16.4)	32 (53.3)

The majority of the fractures were type C. In the dorsal group more than half were type C3 fractures

The preoperative radial inclination for the whole population was 15.2° (SD 9.2°) and the volar tilt was −13.0° (SD 17.7°). The preoperative ulna variance was 1.39 mm (SD 2.96 mm). The postoperative values were 22.1° (SD 4.8°) for radial inclination and 8.6° (SD 6.4°) for volar tilt; ulnar variance was −0.35 mm (SD 1.95 mm). The assessment of the reduction was further analyzed separately for the volar and the dorsal groups (Table 2). The difference in the postoperative reduction of the fractures between the two groups was statistically significant in both planes, with palmar plating achieving better results for radial inclination, and dorsal plating for palmar tilt and ulnar variance. However, the absolute difference was no more than two degrees; a nearly anatomic reduction was achieved for both approaches. The observed statistically significant difference in the palmar tilt between the two groups remained even after comparison of type C fractures only (volar tilt: palmar group (*n* = 112), 7.7° (SD 6.6°)/dorsal group (*n* = 52), 10.5° (SD 6.2°); *p* = 0.011). In the comparison of the radial inclination of type C fractures only, the difference between the two groups was no longer statistically significant. However, a tendency to higher values in the palmar group was observed (palmar group (*n* = 112): 22.4° (SD 4.6°)/dorsal group (*n* = 52): 20.9° (SD 4.7°); *p* = 0.055). The mean operative time remained statistically significantly longer for the dorsal group, even in the comparison of the AO type C fractures only (palmar group: 105.2 min (SD 49.5 min)/dorsal group: 122.6 min (SD 47.3 min); *p* = 0.034). In the palmar group no correlation was seen between fracture severity according to AO classification and postoperative radiological outcome. In the dorsal group a weak negative correlation between fracture severity and radial inclination was observed (*p* = 0.004; Spearman’s *ρ* −0.376).

**Table 2:** Pre- and postoperative radiological parameters

Reduction parameters	Groups			
	Pre-operative palmar	Pre-operative dorsal	Post-operative palmar	Post-operative dorsal
Radial inclination	15.1° (SD 8.7°)	15.7° (SD 10.8)	22.3° (SD 4.7°)	21.1° (SD 5°)
*p*	NS		0.044	
Volar tilt	−13.4° (SD 1.2°)	−12.8° (SD 2.1°)	8.1° (SD 6.3°)	10.1° (SD 6.4°)
*p*	NS		0.01	
Ulnar variance (mm)	1.63 (SD 2.72)	0.88 (SD 3.12)	−0.2 (SD 1.9)	−0.8 (SD 2.3)
*p*	0.001		0.001	

Eighteen complications were recorded overall (Table 3). In the majority of cases (13/18) the complications occurred in type C fractures. In the palmar group, eight complications occurred (3.6 %), while in the dorsal group the incidence was higher (ten cases, 16.7 %). The difference in the incidence was statistically significant (*p* < 0.001). This difference between the two groups remained statistically significant even after comparison of type C fractures only (*p* < 0.001). No significant difference was observed in complication rates with regard to plate type (2.4 mm/3.5 mm).

**Table 3:** Complication rates in the study population and in the subgroups

Complications (*n* = 18/6.3 %)	Groups	
	Palmar (*n* = 8)	Dorsal (*n* = 10)
Pain/swelling	5	5
Tenosynovitis	0	2
EPL rupture	0	1
Intra-articular screw	0	1
Fragment displacement	1	0
Carpal tunnel syndrome	1	0
Re-fracture	1	0
CRPS	0	1
Incidence	3.6 %	16.7 %
*p*	<0.001	

Implant removal was performed in 25 cases in the overall study population (8.8 %)—15 were performed in the palmar group (6.7 %) and 10 in the dorsal group (16.7 %). The difference in the incidence was statistically significant (*p* < 0.01). The indication for implant removal was implant-associated problems (pain or persistent swelling located above the plate) in ten cases (five in the volar group, five in the dorsal group), as well as tenosynovitis of the EPL tendon in two cases and one intra-articular screw in the dorsal group. In the remaining cases the implant removal was initiated after patient request.

## Discussion

As the trend currently leads towards palmar plating [6], the need for dorsal plating is fundamentally questioned. Nowadays, >30 different types of locking plates are available on the world market, with most of them being palmar plates. Novel implants with more screw placement modalities have been introduced; however, the importance of such features remains unconfirmed [7].

In the present study, the postoperative reduction of radial inclination, palmar tilt and ulnar variance in both groups was almost anatomic; this was also seen in previous studies [8, 9]. The absolute value of the observed statistically significant difference between the two groups postoperatively was minimal. Radial inclination seems to be better reduced through a palmar approach; the observed negative correlation between fracture severity according to AO and radial inclination in the dorsal group underlines this fact. However, palmar tilt and ulnar variance were better restored through a dorsal approach. The observed differences between the groups remained, even after comparison of type C fractures only. This fact underlines the above-mentioned differences in the surgical outcome between the two approaches. Nonetheless, it is questionable whether such small absolute differences are of clinical relevance.

The reported complication rates of palmar versus dorsal locking plates in the literature remain contradictory. While several studies report higher complication rates after palmar locked plating [9–11], others show no difference between the two approaches [12, 13]. Making the situation even more confusing, other studies report higher complication rates after dorsal plating [3, 14]. In the present study, the complication rates encountered in the dorsal group were significantly higher. This difference remained statistically significant even after comparison of the type C fractures only; however, this could be attributed to the more demanding surgical technique of dorsal plating with possible devascularization of soft tissues and bony structures, as well as the iatrogenic tendon injury with the addition of longer operative time. Additionally, the positioning of dorsal plates right under the tendon sheaths can further irritate the tendons postoperatively and lead to implant-associated pain. While implant removal rates in the dorsal group were also significantly higher, it was interesting that in almost half of the cases, implant removal was initiated by the patients themselves, even in the absence of objective impairment. The problem of foreign body feeling has not yet been overcome, even after plate design optimization [15]. We generally do not advocate an implant removal unless hardware-associated tendon pathology or functional impairment is present.

The high incidence of tendon ruptures after locked plating reported in the literature, even after palmar osteosynthesis due to oversized screws, was not confirmed in our study. This is in accordance with other studies [16]. In most cases of volar plating, tendon irritations seem to derive from technical errors and oversized screws [17]. The problem of oversized screws may derive from the traditional idea that bicortical screw purchase is needed for plate fixation; this is not the case for internal fixators such as locking plates. As a recent biomechanical study showed, a screw length of 75 % of the anteroposterior cortical distance can result in sufficient buttressing of the joint surface [18]. Nevertheless, if dorsal key fragments need to be fixed, meticulous fluoroscopical control using dynamic and dorsal tangential views can avoid screw oversizing [19, 20]. Tenosynovitis of the flexor was not observed in the present study; however, this could be attributed to the the smaller plate profile and its shape variety (L-, T-plates) with implant placement proximal to the watershed-line [21–23].

The main advantage of dorsal plating is the fact that centrally depressed and dorsal articular fragments can be directly addressed and anatomically reduced; this is not possible through a palmar approach, at least not to that extent. This point finds its implementation mainly in the treatment of complex multifragmentary intra-articular type C3 fractures, or of special fracture types, such as Barton fractures. The question that arises is whether a perfect reduction is needed in every case, especially if it could be associated with higher complication rates. It has been shown that in older patients a certain degree of loss of anatomic reduction can be tolerated to a certain extent, without affecting the subjective final outcome [24, 25]. For younger patients, however, this may not be the case and until proven otherwise, an anatomic reduction in order to minimize the risk of post-traumatic arthritis should be one of the main goals of operative treatment in that patient group.

The final aspect that should be taken into consideration is the biomechanical behavior of different types of plates. Several studies have confirmed the biomechanical superiority of locking plates over conventional plates [8, 26]. This has also contributed to the increasing number of fractures treated with palmar locking plates. However, the biomechanical equality of palmar versus dorsal locking plates still remains debatable. While several studies show no biomechanical differences between the two implants [27], others still advocate the biomechanical superiority of the dorsal plates, which are supposed to be stiffer and stronger [28]. As long as no undisputable proof of the biomechanical equality between palmar and dorsal locking plates exists, the use of dorsal locking implants for the treatment of fractures in high risk for secondary loss of reduction may be taken into consideration.

In conclusion, the present study showed that regardless of fracture severity, an adequate reduction of distal radius fractures is possible through both surgical approaches in the vast majority of the cases. The higher complication and implant removal rates of dorsal locking plates, as well as the longer operative time needed, are factors in favor of palmar locking plates; therefore, we advocate its use for the vast majority of fractures. However, in cases of complex multifragmentary articular fractures, where no compromise in postoperative reduction can be accepted, and as long as the undisputable biomechanical equality of palmar and dorsal plating remains unproven, dorsal locking plates can still be considered as a treatment option in special cases.

## References

[1] Smektala R, Endres HG, Dasch B, Bonnaire F, Trampisch HJ, Pientka L (2009). Quality of care after distal radius fracture in Germany. Results of a fracture register of 1,201 elderly patients. Unfallchirurg.

[2] Liporace FA, Adams MR, Capo JT, Koval KJ (2009). Distal radius fractures. J Orthop Trauma.

[3] Rein S, Schikore H, Schneiders W, Amlang M, Zwipp H (2007). Results of dorsal or volar plate fixation of AO type C3 distal radius fractures: a retrospective study. J Hand Surg Am.

[4] Jupiter JB, Marent-Huber M (2010). Operative management of distal radial fractures with 2.4-millimeter locking plates: a multicenter prospective case series. Surgical technique. J Bone Joint Surg Am.

[5] Kreder HJ, Hanel DP, McKee M, Jupiter J, McGillivary G, Swiontkowski MF (1996). X-ray film measurements for healed distal radius fractures. J Hand Surg Am.

[6] Maschke SD, Evans PJ, Schub D, Drake R, Lawton JN (2007). Radiographic evaluation of dorsal screw penetration after volar fixed-angle plating of the distal radius: a cadaveric study. Hand (N Y).

[7] Drobetz H, Schueller M, Tschegg EK, Heal C, Redl H, Muller R (2011). Influence of screw diameter and number on reduction loss after plating of distal radius fractures. ANZ J Surg.

[8] Konstantinidis L, Helwig P, Strohm PC, Hirschmuller A, Kron P, Sudkamp NP (2010). Clinical and radiological outcomes after stabilisation of complex intra-articular fractures of the distal radius with the volar 2.4 mm LCP. Arch Orthop Trauma Surg.

[9] Matschke S, Wentzensen A, Ring D, Marent-Huber M, Audige L, Jupiter JB (2011). Comparison of angle stable plate fixation approaches for distal radius fractures. Injury.

[10] Knight D, Hajducka C, Will E, McQueen M (2010). Locked volar plating for unstable distal radial fractures: clinical and radiological outcomes. Injury.

[11] Yu YR, Makhni MC, Tabrizi S, Rozental TD, Mundanthanam G, Day CS (2011). Complications of low-profile dorsal versus volar locking plates in the distal radius: a comparative study. J Hand Surg Am.

[12] Zettl RP, Clauberg E, Nast-Kolb D, Ruchholtz S, Kuhne CA (2009). Volar locking compression plating versus dorsal plating for fractures of the distal radius: a prospective, randomized study. Unfallchirurg.

[13] Chou YC, Chen AC, Chen CY, Hsu YH, Wu CC (2011). Dorsal and volar 2.4-mm titanium locking plate fixation for AO type C3 dorsally comminuted distal radius fractures. J Hand Surg Am.

[14] Arora R, Lutz M, Zimmermann R, Krappinger D, Gabl M, Pechlaner S (2007). Limits of palmar locking-plate osteosynthesis of unstable distal radius fractures. Handchir Mikrochir Plast Chir.

[15] Kwan K, Lau TW, Leung F (2011). Operative treatment of distal radial fractures with locking plate system-a prospective study. Int Orthop.

[16] Hakimi M, Jungbluth P, Windolf J, Wild M (2010). Functional results and complications following locking palmar plating on the distal radius: a retrospective study. J Hand Surg Eur.

[17] Tarallo L, Mugnai R, Zambianchi F, Adani R, Catani F (2013). Volar plate fixation for the treatment of distal radius fractures: analysis of adverse events. J Orthop Trauma.

[18] Wall LB, Brodt MD, Silva MJ, Boyer MI, Calfee RP (2012). The effects of screw length on stability of simulated osteoporotic distal radius fractures fixed with volar locking plates. J Hand Surg Am.

[19] Sugun TS, Karabay N, Gurbuz Y, Ozaksar K, Toros T, Kayalar M (2011). Screw prominences related to palmar locking plating of distal radius. J Hand Surg Eur.

[20] Ozer K, Toker S (2011). Dorsal tangential view of the wrist to detect screw penetration to the dorsal cortex of the distal radius after volar fixed-angle plating. Hand (N Y).

[21] Jupiter JB, Marent-Huber M (2009). Operative management of distal radial fractures with 2.4-millimeter locking plates. A multicenter prospective case series. J Bone Joint Surg Am.

[22] Soong M, van Leerdam R, Guitton TG, Got C, Katarincic J, Ring D (2011). Fracture of the distal radius: risk factors for complications after locked volar plate fixation. J Hand Surg Am.

[23] Asadollahi S, Keith PP (2013). Flexor tendon injuries following plate fixation of distal radius fractures: a systematic review of the literature. J Orthop Traumatol.

[24] Gruber G, Zacherl M, Giessauf C, Glehr M, Fuerst F, Liebmann W, Gruber K, Bernhardt GA (2010). Quality of life after volar plate fixation of articular fractures of the distal part of the radius. J Bone Joint Surg Am.

[25] Egol KA, Walsh M, Romo-Cardoso S, Dorsky S, Paksima N (2010). Distal radial fractures in the elderly: operative compared with nonoperative treatment. J Bone Joint Surg Am.

[26] Levin SM, Nelson CO, Botts JD, Teplitz GA, Kwon Y, Serra-Hsu F (2008). Biomechanical evaluation of volar locking plates for distal radius fractures. Hand (N Y).

[27] McCall TA, Conrad B, Badman B, Wright T (2007). Volar versus dorsal fixed-angle fixation of dorsally unstable extra-articular distal radius fractures: a biomechanic study. J Hand Surg Am.

[28] Blythe M, Stoffel K, Jarrett P, Kuster M (2006). Volar versus dorsal locking plates with and without radial styloid locking plates for the fixation of dorsally comminuted distal radius fractures: A biomechanical study in cadavers. J Hand Surg Am.

